# Flowering plant embryos: How did we end up here?

**DOI:** 10.1007/s00497-021-00427-y

**Published:** 2021-07-27

**Authors:** Stefan A. Rensing, Dolf Weijers

**Affiliations:** 1 Plant Cell Biology, Department of Biology, University of Marburg, Marburg, Germany; 2 BIOSS Centre for Biological Signalling Studies, University of Freiburg, Freiburg, Germany; 3 Laboratory of Biochemistry, Wageningen University, Stippeneng 4, 6708WE Wageningen, The Netherlands

## Abstract

The seeds of flowering plants are sexually produced propagules that ensure dispersal and resilience of the next generation. Seeds harbor embryos, three dimensional structures that are often miniatures of the adult plant in terms of general structure and primordial organs. In addition, embryos contain the meristems that give rise to post-embryonically generated structures. However common, flowering plant embryos are an evolutionary derived state. Flowering plants are part of a much larger group of embryo-bearing plants, aptly termed Embryophyta. A key question is what evolutionary trajectory led to the emergence of flowering plant embryos. In this opinion, we deconstruct the flowering plant embryo and describe the current state of knowledge of embryos in other plant lineages. While we are far yet from understanding the ancestral state of plant embryogenesis, we argue what current knowledge may suggest and how the knowledge gaps may be closed.

## Introduction

When asked the question “What is a plant embryo?”, one may intuitively think of those that we meet in daily life: the seeds of flowering plants. This is understandable, since much of our diet is made from flowering plant seeds (think of beans, nuts or cereal grains). These seeds contain a mature embryo that generates a miniature form of the adult individual after its germination. This miniature form, the seedling, then goes on to form the adult plant. However, when considering the evolutionary history of plant embryogenesis, it should be clear that this form of embryogenesis is a highly derived state, brought forward by a number of innovations that occurred during the long and rich history of flowering plant evolution.

In this opinion, we will deconstruct flowering plant embryogenesis into these innovations and drill down to the humble beginnings of plant embryogenesis. Insight into the constituent steps in the evolution of plant embryogenesis not only gives a rich context for understanding the unique properties of plant embryos, but also offers a framework for discussing the conservation of key principles and the variation of the process among plant groups.

## Deconstructing the flowering plant seed and embryo

Within flowering plants (angiosperms), seeds are formed within flowers and are housed in specialized structures for protection and later dispersal—the ***fruit*** (reviewed in Zúñiga-Mayo et al. 2019). The ***seed*** itself is an interesting, hybrid structure, that is composed of three genetically separate tissues: the embryo—a diploid structure and product of fertilization of the haploid male and female gametes); the endosperm—a triploid fertilization product that nourishes the embryo; and the seed coat—a diploid, maternal tissue that protects and encapsulates both embryo and endosperm. Conceptually, the endosperm has analogies to the mammalian placenta. In contrast to the placenta, that consists of both zygotic and maternal tissues, the endosperm is entirely a product of fertilization. Through seed coat and endosperm, the flowering plant embryo is nourished by the sporophyte (reviewed in Baroux and Grossniklaus 2019), while the bryophyte embryo, as well as the engulfing sporophyte, is nourished (primarily via sucrose transport) by the gametophyte (Regmi et al. 2017). The embryo itself is a miniature version of the plant, with one (monocots) or two (dicots) cotyledons (or scutellum in monocots), an embryonic stem and root, and meristems for shoot and root systems (reviewed in Dresselhaus and Jürgens 2021). These meristems are generally ***indeterminate*** meaning that they can continuously produce aerial or root tissues and organs, while maintaining their own structure (reviewed in Umeda et al. 2021). The partitioning of the embryo in different organs—and in different tissues (epidermal, ground tissue, vascular)—underscores the notion that a ***pattern formation*** process, in which cells specialize in an ordered pattern, is an intrinsic part of embryogenesis (reviewed in Palovaara et al. 2016). The embryo is multicellular, consisting of a large number of diploid cells derived from ***mitotic divisions*** of the zygote. Thus, sustained cell divisions are an important part of embryogenesis. Given that the embryo is a ***fertilization*** product, it is formed by fusion of cells from maternal and paternal parental organs (reviewed in Sharma et al. 2021). Lastly, the maternal and paternal cells that generate the zygote are specialized, haploid sexual cells—***gametes***—that have to be generated in each parent for fertilization to be possible.

**Fig. 1:**
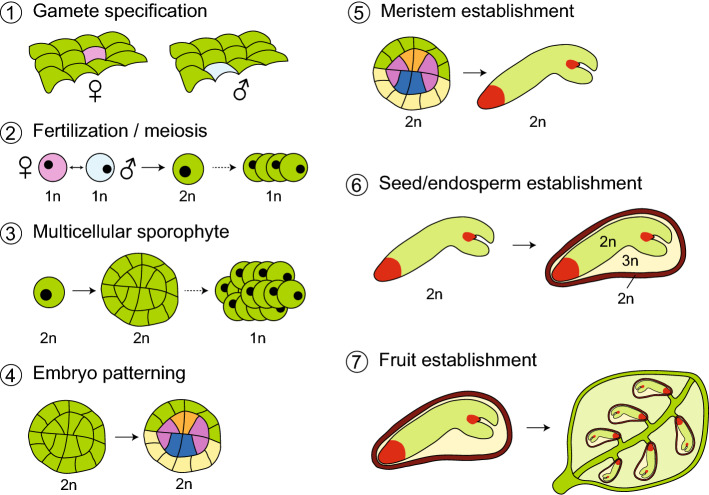
Innovations in sexual reproduction in land plants Illustrations of seven discrete steps in the evolution of sexual plant reproduction. **1** A key first step is the selection of specification of gametes or gametic cells (pink and blue) from a field of non-gamete cells. From the gametic cells, sexual organs may arise that generate gametes. The gametes need to be compatible for biparental mating, here indicated with male and female signs. **2** Next, mating-compatible haploid (1n) gametes need to fuse through fertilization (or conjugation) to give rise to a diploid (2n) zygote that then undergoes meiosis and generates haploid progeny. **3** A third innovation is the separation of fertilization and meiosis by a multicellular, diploid, sporophytic phase that spawns a larger number of meiotic cells per zygote (see Rensing 2016 for comparison of life cycles). **4** Rather than being of uniform identity, the multicellular sporophyte (embryo) can be partitioned into a pattern of functionally distinct cell types (here marked by different colors). **5** A key innovation is the establishment of indeterminate meristems (red; in seed plants) within the embryo. These can generate shoot or root tissue for prolonged periods. The timing of activation of such meristems, the degree of indeterminacy, and whether there is a pause between embryonic meristem establishment and meristem activity are all features that can differ between plant groups and species. **6** The evolution of the seed as an embryo-bearing capsule generated a protective layer of maternal origin (brown, diploid). Many seed plants feature double fertilization, which generates a nurturing endosperm (triploid, 3n) in addition to the embryo. In bryophytes, the spore capsule or the haploid spores contained within are the propagules. **7** The evolution of fruits as seed-bearing structures offered further protective mechanisms, as well as additional instruments for seed dispersal

With the above deconstruction, it should be clear that the development of flowering plant embryos is in fact the outcome of a long series of individual steps [gametes–fertilization–mitotic divisions–pattern formation–indeterminacy–seed–fruit; Fig. 1], each of which must have emerged at one time during plant evolution. In the following sections, we will discuss each of the steps that pertain to embryo development (excluding the seed and fruit) and place their appearance in the context of plant evolution.

## The formation of haploid gametes and fertilization

Within the plant kingdom, sexual reproduction using egg and sperm cells is found in all groups of land plants and three lineages of streptophyte algae, the Zygnematophyceae, Coleochaetophyceae, and Charophyceae (Rensing et al. 2020). This group is collectively referred to as Phragmoplastophyta, and it appears that this mode of reproduction evolved in the most recent common ancestor (MRCA) of this group. A recent analysis of a key regulator of sperm cell differentiation in the flowering plant *Arabidopsis thaliana*, the transcription factor DUO1, revealed that the regulation of sperm cell development may be ancestral among the Phragmoplastophyta (Higo et al. 2018). While the genetic networks downstream of this factor have evolved in different directions, along with the exact modes of male gamete development (e.g., sperm motility), the same factor appears to contribute to sperm function in Arabidopsis and the liverwort *Marchantia polymorpha*, and domain swaps suggest a route for innovations in this regulator within the Phragmoplastophyta.

Interestingly, while the Phragmoplastophyta share the mode of sexual reproduction, and thus the alternation between haploid (gametophyte) and diploid (sporophyte) generations, the strategies are not uniform. It appears that sperm motility evolved early, but this trait has been lost several times independently (Higo et al. 2018; Meyberg et al. 2020). In those species where the trait was lost, gametes either conjugate (in conjugating Zygnematophycean algae), or the sperm cells are carried to the female gamete by a pollen tube. The process of gamete interaction seems to have co-opted a deeply conserved fusogen. The HAP2/GCS1 protein is distributed widely in eukaryotes (Hirai et al. 2008; Liu et al. 2008) and was identified for its role in gamete fusion in Arabidopsis (Mori et al. 2006; von Besser et al. 2006). The protein resembles viral fusogen proteins (Valansi et al. 2017), so called for their ability to promote membrane fusion. A recent structural analysis of the *Chlamydomonas reinhardtii* HAP2 confirmed homology of this ancestral eukaryotic protein (Fédry et al. 2017). Thus, this membrane fusion protein has been recruited into gamete fusion in a range of eukaryotes, including the Phragmoplastophyta.

Research into the regulation of gamete specification in Arabidopsis has identified several factors that contribute to setting apart gametes from sporophytic cells (e.g., Mendes et al. 2020). Some factors have recently been shown to be conserved between bryophytes and flowering plants, and hence probably carry out a conserved function in all Embryophyta. Examples include BELL/KNOX (homeodomain transcription factor, HD TF) interactions that control the haploid to diploid transition in the moss *Physcomitrium* (Horst et al. 2016; Ortiz-Ramirez et al. 2017), or formation and maturation of gametangia in *Physcomitrium* and *Marchantia* that are controlled via transcriptional and epigenetic switches that are also involved in seed plant germ line development (Yamaoka et al. 2018, Genau et al. 2021; Hisanaga et al. 2019). It is almost entirely unknown how gametes are set aside (during conjugation) or specified (motile sperm) in algal sister lineages to the land plants. Molecular and genetic analysis of such species will likely bring such insights. With an increasing number of genomes becoming available in this group (Nishiyama et al. 2018; Cheng et al. 2019; Jiao et al. 2020) and with reports of genetic transformation (Abe et al. 2011; Sørensen et al. 2014; Regensdorff et al. 2018), the roots of this most fundamental of processes in plant sexual reproduction will hopefully soon be explored.

## Development of a multicellular sporophyte

While all Phragmoplastophyta share sexual reproduction through specialized gametes, there is an important distinction between algal and land plant species. The streptophyte algae are haplonts, meaning that the zygote is the only diploid cell throughout the life cycle (see Rensing 2016 for review of life cycles). In contrast, all land plants are haplodiplonts, meaning that both generations are multicellular. This property is at the core of plant embryogenesis, where a multicellular fertilization product is formed. It is for this reason that land plants are collectively referred to as Embryophytes. The distinction between the algal and land plant modes is in whether fertilization is immediately followed by meiosis (algae), or by mitotic divisions (land plants). In this context, the land plant mode can be considered one of postponed meiosis, where gametes are formed only later, and within specialized niches on a more elaborate plant body. Thus, the evolution of the multicellular embryo must have been accompanied by a change in the way divisions are controlled. The triggers and mechanisms of mitotic cell division have been studied in detail in Arabidopsis and other flowering plants (Gutierrez 2016), and it will be interesting to see how these differ between land plant embryos and algal zygotes. However, the triggers of meiotic cell division are not understood in as much detail. In Arabidopsis, several mutants have been identified in which the zygote arrests (Guo et al. 2016; Hou et al. 2021). Given that both gametes in Arabidopsis involve haploid cell divisions, this suggests that mitotic divisions in gametophyte and sporophyte involve different factors. Are these arrested zygotes functionally comparable to algal zygotes? Once the differences between mitotic and meiotic division are understood in sufficient detail, one can start to address this question.

Within the embryophytes, the embryo can serve one of two purposes. The embryo either is a transient, sporophytic stage during which proliferative divisions occur to increase the number of cells that will then commit to meiosis and generate a large number of haploid spores. In such embryos, there is limited functional specialization among cells, and most terminate in meiosis. Alternatively, the embryo undergoes functional differentiation of different cell types, such as conductive vascular cells, ground tissue, and epidermis. The former type is found in bryophytes, where for example in *Marchantia polymorpha*, limited cell differentiation occurs in the embryo (Shimamura 2016). In general, tracheophytes (vascular plants, including seed and flowering plants) have more extensive cell differentiation in the embryo (Palovaara et al. 2016). The separation is not absolute, since substantial differentiation is found in moss embryos (e.g., *Physcomitrium*; Landberg et al. 2013, Hiss et al. 2017). It is therefore not clear what the ancestral state of pattern formation was in the ancestor of all land plants. It is likely that the ancestral state of the Embryophyta sporophyte was substantially complex and that the liverwort lineage lost some of this complexity.

One clear difference between tracheophyte and bryophyte embryos is the separation of photosynthetic (leafy) and anchoring (rooting) functions to defined domains. Thus, the establishment of the embryo found in flowering plants, from the zygote, must have involved at least two decisive innovations: promotion of mitotic divisions and the establishment of cellular specialization.

Nonetheless, there is clearly a larger degree of tissue complexity in Tracheophyta embryos, which has been studied in substantial detail in the dicot Arabidopis, as well as the monocots rice and maize (Dresselhaus and Jürgens, 2021). In addition, several studies have focused on cellular pattern formation in gymnosperms (Palovaara et al. 2010; Alvarez et al. 2018). From these studies, it is clear that a small number of genes marks potentially homologous domains or cell types across gymnosperm and angiosperm embryos. From a recent transcriptomic comparison between Arabidopsis and *Brachypodium distachyon* (a monocot) embryos, it appears that many more regulators may follow a similar temporal expression pattern across angiosperms (Hao et al. 2021). However, a major limitation in making inferences about the ancestral state of tracheophyte embryogenesis is that detailed knowledge is available in a single species only. It is entirely possible that Arabidopsis is not representative of embryogenesis in tracheophytes in its mode of pattern formation. Thus, a clear future mission should be the in-depth analysis of embryo patterning processes and regulators in a broader range of tracheophytes. This should include species at key phylogenetic positions, such as angiosperms that are sister to the core lineages (e.g., *Amborella trichopoda*), gymnosperms (e.g., *Ginkgo biloba*), ferns (e.g., *Ceratopteris richardii*) and horsetails (e.g., *Equisetum* genus). Clearly, this will be challenging given that none of these are yet facile experimental and genetic model organisms.

## The establishment of indeterminate meristems

Whether the embryo is truly a miniature version of the adult plant, and if it can directly sustain post-embryonic organogenesis, depends on whether indeterminate meristems are initiated in the embryo. Meristems in land plants come in different types: In seed plants, shoot meristems have a characteristic tunica/corpus structure, with well-defined tissue layers, while root meristems also have a clear, yet distinct organization of cell and tissue types. In such meristems, cell division activity and “stemness” are clearly arranged, and new primordia bud off the flanks of the meristem (shoot), or are produced by cells that pass through the meristem (root). The molecular architecture of the regulation underlying the shoot meristem type has been studied in great detail, and involves HD TFs, small peptide signals and their membrane receptors, among many other factors (Somssich et al. 2016). Neither the moss *Physcomitrium patens* nor the fern *Ceratopteris richardii* feature this meristem type, but instead have one or two apical cells in their shoot tip that alternate division plane to give rise to a subtending mass of cells that then form the organs (Plackett et al. 2015; Véron et al. 2021). Yet despite these structural differences, there are homologies in the molecular components of their regulation. Mutants of the Physcomitrium orthologs of the Arabidopsis CLAVATA peptide and receptors show defects in apical cell divisions (Whitewoods et al. 2018). Likewise, the *Marchantia polymorpha* orthologues are involved in stem cell function in the thallus (Hirakawa et al. 2020). It should be noted that in both these cases, it is the haploid gametophyte in which CLAVATA function is apparent, while in Arabidopsis this function is restricted to the sporophyte. This may mean that the gametophyte and sporophyte use the same (or similar) genetic regulators and networks to control development. This notion is not easy to test, given that the gametophytes are strongly reduced in flowering plants, where CLAVATA functions in the sporophyte are best studied. The major difference between bryophytes and tracheophytes in this context would then be the reduced gametophytic lifespan and protracted sporophytic stage in tracheophytes. Genetic analysis of TCP transcription factors supports this notion: These proteins repress branching in flowering plants (sporophytes). Loss-of-function of a *Physcomitrium* TCP gene leads to branching of the sporophyte. This suggest an ancestral function of these genes in repressing sporophytic branching (Ortiz-Ramírez et al. 2016). Similarly, the polycomb repressive complex 2 (that deposits histone 3 K27 trimethylation) represses the diploid body plan, and loss-of-function may result in branched sporophytes (Okano et al. 2009). Lastly, it should again be noted that the distinction between indeterminate sporophytes in Tracheophytae and determine sporophytes in bryophytes is not absolute: there is limited indeterminacy in the moss and hornwort sporophytes (the latter grow from a meristem-like zone at their base). While this meristematic activity is not comparable to that found in vascular plant sporophytes, it does imply that the genetic program for indeterminate sporophytic growth might have been present in the ancestor to all land plants. Future genetic studies across land plants should reveal whether the same molecular networks operate in all species, and what shortcuts, abbreviations, and extensions have given rise to the unique patterns observed today.

## Concluding remarks

For decades, the well-studied Arabidopsis embryo has been considered typical for plant embryos. However, studies in other flowering plants have shown that drastic differences in embryogenesis have resulted even in the time since the flowering plant radiation. Extant plant lineages that represent evolutionary divergence times that far exceed this event have recently been studied in order to understand the different flavors of plant embryogenesis, and how it evolved. By comparing embryos and embryogenesis across, e.g., flowering plants, ferns, and bryophytes we can infer the evolution of embryogenesis. The ultimate goal is to define the ancestral state as well as character evolution that occurred for the past half billion years and resulted in drastically different structures, such as flowering plant seeds and moss spore capsules. Yet, despite all differences, some key features of the molecular regulation of embryogenesis, such as the involvement of PRC2 complexes and HD-TALE transcription factors, have been conserved. Future studies of a diverse set of plant lineages, and of streptophyte algae representing the sister lineages to land plants, shall further our understanding of how this enigmatic structure and its control evolves.
